# Extended-spectrum beta-lactamase-producing *Klebsiella pneumoniae* and risk factors associated with high total bacterial count in bulk tank milk from dairy farms in Colombia

**DOI:** 10.1007/s42770-024-01396-w

**Published:** 2024-06-14

**Authors:** Ángela-Sofía Ágredo Campos, Ömer Akineden, Jorge A. Fernández-Silva, Nicolás F. Ramírez-Vásquez

**Affiliations:** 1 Grupo Centauro, Escuela de Medicina Veterinaria, Facultad de Ciencias Agrarias, Universidad de Antioquia UdeA, Calle 70 No. 52-21, Medellín, Colombia; 2 Dairy Sciences, Institute of Veterinary Food Science, Justus-Liebig-University Giessen, Ludwigstrasse 21, 35390 Giessen, Germany

**Keywords:** Bulk tank milk, Total bacterial count, Antimicrobial resistance, ESBL

## Abstract

The objective of the study was to evaluate the frequency and genetic characteristics of ESBL-producing *Escherichia coli* and *Klebsiella* spp. and the risk factors associated with a high total bacterial count in bulk tank milk samples of dairy farms in three municipalities of the Antioquia Department, Colombia. Fifteen samples were positive for *E. coli* and *Klebsiella* spp. Subsequent analysis of the 16 S rRNA gene sequences confirmed these isolates included *E. coli* (*n* = 3), *K. oxytoca* (*n* = 11), and *K. pneumoniae* (*n* = 1). None of the isolates was positive for ESBL identification by phenotypic methods, but the only the isolate of *K. pneumoniae* was positive for the *bla*SHV61 gene by sequence analysis. The antibiotic susceptibility evaluation for all *Klebsiella* spp. isolates identified resistance to fosfomycin (50%; 6/12) and ampicillin (100%; 12/12). While most of the herds maintain adequate hygienic quality, specific risk factors such as having more than 60 milking cows, frequent changes in milkers, milking in paddocks, and using a chlorinated product for pre-dipping have been identified as associated with a high total bacterial count > 100,000 CFU/mL in bulk tank milk. However, certain variables including the milker being the owner of the animals and the proper washing and disinfection of the milking machine contribute to maintain a high level of hygiene and quality in the raw milk stored in the tanks. In conclusion, the frequency of ESBL producers was relatively low, with only *K. pneumoniae* testing positive for the *bla*SHV ESBL type. The presence of these bacteria in milk tanks represents a potential risk to public health for consumers of raw milk and its derivatives.

## Introduction

Raw bovine milk may contain pathogenic microorganisms that originate from multiple sources of contamination. Due to contaminants introduced through cow udder teats, milking system, and farm environment, the final composition of the microorganisms in bulk tank milk is highly diverse [1]. The hygienic quality of bulk tank milk in Colombia is evaluated by estimating the total bacterial count [2]. A high total bacterial value could indicate contamination mainly with environmental bacteria due to poor hygiene measures at the time of milking, contaminated milking equipment, and insufficient or inadequate cooling during transportation and storage [3–5]. In addition to the safety concerns associated with raw milk, an increase in the total bacterial count can also adversely affect its organoleptic and nutritional properties, potentially reducing the selling price or rendering the product unfit for human consumption. Moreover, tank milk samples can also be an important and valuable tool for estimating the individual farm situation concerning to overall bacteriological quality of the milk and pathogenic microorganisms, especially antibiotic resistance situations at the herd level in dairy farms [6, 7]. Due to the growing worldwide problem of resistance to broad-spectrum antibiotics such as cephalosporins among *Enterobacteriaceae*, studies have increased in recent years, particularly focusing on foods of animal origin and dairy farms as possible reservoirs of resistant bacteria that produce extended-spectrum beta-lactamases (ESBLs). Most of these studies were conducted with *Escherichia coli* and *Klebsiella* spp., which are generally considered valuable indicators of antibiotic resistance due to their wide entry into the food chain and environment, as well as their potential for gene exchange between humans and animals. Several studies have identified the presence of ESBL-producing *Enterobacteriaceae* in bovine milk with clinical mastitis [8, 9]. However, only limited data are available on the presence of ESBL-producing *Enterobacteriaceae*, especially *E. coli* and *Klebsiella* spp., in bulk tank milk [10–12]. In Colombia, Vásquez-Jaramillo et al. (2017) first reported in a previous local study the presence of ESBL-carrying *Enterobacteriaceae* in bulk tank milk samples of dairy herds from a municipality in Antioquia. This suggests that ESBLs are circulating among *Enterobacteriaceae* in the country´s dairy herds, necessitating the development of larger epidemiological studies that can provide more detailed and accurate information on this phenomenon.

Therefore, this study aimed to estimate the frequency and genetic characteristics of ESBL-producing *E. coli* and *Klebsiella* spp. in bulk tank milk samples from dairy farms in three geographically different municipalities of the Antioquia Department, Colombia. Additionally, it aimed to identify the risk factors associated with a high total bacterial count.

## Materials and methods

### Herds and samples

Information on the dairy herds sampled and the procedure for collecting and handling bulk-tank milk samples for microbiological analysis was previously published by Ágredo-Campos et al., 2023. A total of 150 dairy herds were randomly selected from three municipalities (Santa Rosa de Osos, San Pedro de los Milagros, and Entrerríos) in northern Antioquia Department, Colombia, between August 2019 and January 2020. The dairy herds were classified based on the number of lactating cows as follows: small herds (< 30 dairy cows), medium herds (30–60 dairy cows), and large herds (> 60 dairy cows). Most of the dairy cows in the herds were of the Holstein breed, and they were grazing with supplementary feed at the time of milking. The milking systems used in the herds were mainly paddock and parlor mechanical milking systems, with a few herds practicing manual milking. For the microbiological analysis and evaluation of the total bacterial count, two milk samples were taken from each herd’s milk tank. These samples were collected in sterile containers containing the Bronopol preservative, following the standards established by the National Mastitis Council [13]. The samples were immediately transported to the Laboratory of Milk Quality at the Faculty of Agricultural Sciences of the Universidad de Antioquia, maintaining a temperature of 4 °C +/- 2 °C during transportation.

### Microbiological analyses

The microbiological analysis for the detection of *E. coli* and *Klebsiella* spp. followed the protocols established by the National Mastitis Council [13]. Briefly, 0.01 mL of milk samples was streaked on MacConkey agar (Merck, Darmstadt, Germany) using a sterile calibrated loop (Biologix® Group Ltd., Shandong, China). After incubation at 37 °C for 24 h, the presumptive *E. coli* and *Klebsiella* spp. colonies grown on the agar were subjected to Gram staining and the oxidase strip test (Merck) for initial identification. Gram-negative and oxidase-negative colonies were further subjected to biochemical tube tests, including triple sugar iron agar, indole, citrate, urea, and lysine tests. Subsequently, the isolates were cultured by streaking on Columbia agar® (BioMérieux, Marcy-l’Étoile, France), supplemented with 5% sheep blood, for further analyses.

### Bacterial confirmation

For the extraction of bacterial DNA, the commercial kit DNeasy UltraClean bacteria (Qiagen, Hilden, Germany) was used following the manufacturer’s instructions. Species-specific identification of isolates was conducted by amplification and sequencing of the 16 S rRNA gene, following the method reported by Kuhnert et al. (1996). PCR products were purified using the QIAquick PCR kit (Qiagen) according to the manufacturer’s recommendations and were subsequently sequenced by Microsynth Sequence Laboratories, Göttingen, Germany under standard conditions. The DNA sequences obtained were then subjected to homology searches using the Basic Local Alignment Search Tool (BLAST) provided by the National Center for Biotechnology Information (NCBI) (available at http://blast.ncbi.nlm.nih.gov/Blast.cgi).

### Phenotypic detection of ESBL

The isolates were screened for ESBL detection using the double-disc synergy test. For this purpose, 1–2 colonies were inoculated in sterile saline solution and adjusted to a density of 0.5 according to the McFarland scale standard (1 × 10^8^ CFU/mL). A sterile swab was dipped into the culture suspension tube, and then a Mueller-Hinton agar plate (BioMérieux) was inoculated by uniformly streaking the swab in three planes at an angle of 60° to each other. Finally, a circle was made to correct any excess on the agar plate. Zones of inhibition were determined for each isolate using antibiotic disks, each containing 30 µg of aztreonam (ATM), cefotaxime (CTX), ceftazidime (CAZ), ceftriaxone (CRO), or cefepime (FEP) (Oxoid, United Kingdom), either alone or in combination with 10 µg of clavulanic acid, with a disc distance of 20 mm. The results were evaluated according to the cut-off points established by the Clinical Laboratory Standard Institute [14]. As negative and positive controls, the susceptible *E. coli* strain ATCC 25,922 and the ESBL-producing *K. pneumoniae* strain ATCC 700,603 were used, respectively.

### Antimicrobial susceptibility testing

Antimicrobial susceptibility testing was carried out using the Vitek® 2 system (BioMérieux) with the AST-N271 according to the manufacturer’s instructions. MIC breakpoints for 17 antibiotics, including ampicillin, ampicillin/sulbactam, cephalothin, cefuroxime, cefotaxime, ceftazidime, ceftriaxone, cefepime, ertapenem, meropenem, amikacin, gentamicin, ciprofloxacin, norfloxacin, fosfomycin, nitrofurantoin, and trimethoprim/sulfamethoxazole, were set according to the Clinical and Laboratory Standards Institute (CLSI) breakpoint tables for the interpretation of MICs and zone diameters (M100: Performance Standards for Antimicrobial Susceptibility test. 30th edition) [14].

### Molecular determination of ESBL

The isolates were subjected to conventional PCR analysis for the identification of β-lactamase (*bla*) genes belonging to the ESBL-subgroup, including TEM, SHV, and CTX-M (CTXM groups 1, 2, 8, 9, and 25), following the methods established by Batchelor et al. (2005) and Pitout et al. (1998). For quality control, positive controls were used, including *K. pneumoniae* strain ATCC 700,603 (harboring *bla*SHV gene) and a strain of *K. pneumoniae* (harboring *bla*CTX-M and *bla*TEM genes), while *E. coli* ATCC 25,922 was used as the negative control for all PCR amplification tests. The resulting amplicons were purified using the PCR Purification Kit (Qiagen), and sequencing was performed at SeqLab in Goettingen, Germany. The obtained sequences were evaluated using the BLAST algorithm available at http://blast.ncbi.nlm.nih.gov/Blast.cgi.

### Detection of shiga toxin-producing *E. coli*

The genes for the two main Shiga toxins, STX1 and STX2, of Shiga Toxin-producing *E. coli* (STEC) were amplified using conventional PCR with primers MK1 and MK2, following previously established protocols by Karch and Meyer (1989).

### Evaluation of total bacterial count

The total bacterial count in samples was determined using the flow cytometry method (ISO 21187:2021/ IDF 196:2021) on the Bactoscan FC + instrument (Foss Electric, Hillerød, Denmark).

### Case definition

A herd was considered positive for the total bacterial count if the value obtained was greater than 100,000 CFU/mL, and it was considered negative if the value was equal to or less than 100,000 CFU/mL, based on the criteria established by the Commission Regulation (CE) No. 853/2004 [15].

### Epidemiological questionnaire

A questionnaire was used to collect data on herd general characteristics, milking management practices, and antibiotic use. The method of application and general findings were previously published [16] and are available upon request. For the determination of the association of general variables and herd management practices with the response variable total bacterial count > 100,000 CFU/mL, the variables “*adequate washing and disinfection of the milking machine*” and “*adequate washing and disinfection of the bulk tank*” were constructed. These processes were considered adequate when the herd had a disinfection protocol for the machine and bulk tank, and this protocol was elaborated by an external provider.

### Statistical analysis

Descriptive statistic was applied for the determination of measures of central tendency and frequency distribution to the variables resistance or susceptibility to the evaluated antibiotics, detection of ESBL-producing bacteria, total bacterial count, and to the variables *adequate washing and disinfection of the milking machine* and *adequate washing and disinfection of the bulk tank.*

For the total bacterial count variable, the geometric and arithmetic mean with its 95% CI and statistical significance were estimated. The latter was estimated using the Mann-Whitney U test since the variables did not follow a normal distribution according to the Kolmogorov-Smirnov test.

For the selection of the variables to be included in the multivariate model, those that in the bivariate analysis had a *p* < 0.25 value or that had been previously reported were used. For the dichotomized total bacterial count variable, binomial regression or Poisson regression was used when the former did not reach convergence. All primary exposures of interest were adjusted for confounding variables and the interaction was assessed by including the term in the model whenever significant. For the selection of the final model, the AIC criterion (Akaike Information Criterion) was used. Adjusted RR with 95%CI was reported and a *p* < 0.05 value was significant. All analyses were performed with Stata 16.0 (StataCorp LLC, Texas, USA).

## Results

### Microbiological isolation and species identification of *Klebsiella* spp. and *E. coli*

After the initial screening of samples, a total number of 15 morphologically different Gram-negative colonies from equal number of bulk-tank milk samples were obtained. For each morphologically distinct type of colony, one isolate representing each bulk-tank milk sample/dairy herd was selected. The isolates were then biochemically identified as *E. coli* (*n* = 3), and *Klebsiella* spp. (*n* = 12). Further, 16 S rRNA gene sequence analysis confirmed these isolates on species level as *E. coli* (*n* = 3), *K. oxytoca* (*n* = 11), and *K. pneumoniae* (*n* = 1) (Table 1).

**Table 1:** Characteristics of *Klebsiella* spp. and *E. coli* isolates from bulk tank milk samples (*n* = 150) of three municipalities of Antioquia (Colombia)

Designation of isolate	No. of Herd	Municipality ^a)^	Species identification by 16 S rRNA sequence analysis				
			16 S rDNA sequencing^b)^	Phylogenetic affiliation	Sequence length (bp)	Identity % (Query Coverage)^c)^	Closest species (NCBI accession number)
*E. coli* #1	2	Santa Rosa	+	*E. coli*	1258	100.00	*E. coli* D16EC0527 (CP088892.1)
*E. coli* #2	13	Entrerrios	+	*E. coli*	1303	99.92	*E. coli* CChl2 (CP091506.1)
*K. oxytoca* #3	42	Santa Rosa	+	*K. oxytoca*	1312	100.00	*K. oxytoca* SHD-1 (GU361112.1)
*K. oxytoca* #4	55	Santa Rosa	+	*K. oxytoca*	1314	99.70	*K. oxytoca* N7 (KM349412.1)
*K. oxytoca* #5	57	Santa Rosa	+	*K. oxytoca*	1309	99.69	*K. oxytoca* N7 (KM349412.1)
*K. oxytoca* #6	75	Santa Rosa	+	*K. oxytoca*	1300	99.38	*K. oxytoca* JCM 1665 (NR112010.1)
*K. oxytoca* #7	77	Entrerríos	+	*K. oxytoca*	1309	99.31	*K. oxytoca* JCM 1665 (NR112010.1)
*K. oxytoca* #8	78	Entrerríos	+	*K. oxytoca*	1291	99.69	*K. oxytoca* JCM 1665 (NR112010.1)
*K. oxytoca* #9	79	Entrerríos	+	*K. oxytoca*	1291	99.69	*K. oxytoca* JCM 1665 (NR112010.1)
*K. oxytoca* #10	87	Entrerríos	+	*K. oxytoca*	1310	99.47	*K. oxytoca* NGB-FR100 (LC049195.1)
*K. pneumoniae* #11	92	Entrerríos	+	*K. pneumoniae*	1315	99.54	*K. pneumoniae* DSM 30,104 (NR_117686.1)
*K. oxytoca* #12	99	Entrerríos	+	*K. oxytoca*	1309	99.77	*K. oxytoca* PS. EI-2 (KM396263.1)
*K. oxytoca* #13	106	Entrerríos	+	*K. oxytoca*	1308	99.62	*K. oxytoca* JCM 1665 (NR112010.1)
*K. oxytoca* #14	131	San Pedro	+	*K. oxytoca*	1258	99.68	*K. oxytoca* KKP 3089 (MT549687.1)
*E. coli* #15	143	San Pedro	+	*E. coli*	1303	99.92	*E. coli* 011D (MN015021.1)

^(a)^ Santa Rosa de Osos: Santa Rosa; San Pedro de los Milagros: San Pedro ^(b)^ Amplification and Sequencing of the 16 S rRNA gene according to Kuhnert et al. (1996); ^c)^obtained by using the Basic Local Alignment Search Tool (BLAST) by the National Center for Biotechnology Information (NCBI) (available at http://blast.ncbi.nlm.nih.gov/Blast.cgi).

### Phenotypic and genotypic detection of ESBL production

None of the *E. coli* and *Klebsiella* spp. isolates were ESBL positive by phenotypic methods. ESBL production, however, was genotypically confirmed only in one isolate (*K. pneumoniae* #11) from the bulk-milk tank from a dairy farm in the municipality Entrerríos (1/150; 0.7%). The isolate *K. pneumoniae* #11 hosted *bla*SHV gene, based on PCR, and subsequently the sequence analysis showed strong homology with *bla*SHV-61 variant. None of the isolates was positive for the *bla*TEM and *bla*CTX-M genes in the genotypic tests (Table 2).

**Table 2:** Antibiotic resistance profiles (Minimum Inhibitory Concentration (MIC) µg/mL) of *E. coli* and *Klebsiella* spp. isolates from bulk tank milk samples (*n* = 150) collected from three municipalities of Antioquia (Colombia)

Isolate	Resistance phenotype	ESBL Genotype^a)^			Susceptibility profile (µg/mL)											
		bla_TEM_	bla_SHV_	bla_CTX−M_	AMP	I	SAM	I	CEF	I	CXM	I	FOF	I	NIT	I
*E. coli* #1	-	-	-	-	≤ 2	S	≤ 2	S	4	S	2	S	≤ 16	S	≤ 16	S
*E. coli* #2	-	-	-	-	≤ 2	S	≤ 2	S	4	S	2	S	≤ 16	S	≤ 16	S
*K. oxytoca #3*	-	-	-	-	8	R	≤ 2	S	≤ 2	S	≤ 1	S	≤ 16	S	32	S
*K. oxytoca* #4	-	-	-	-	16	R	4	S	≤ 2	S	≤ 1	S	128	R	≤ 16	S
*K. oxytoca* #5	-	-	-	-	16	R	4	S	≤ 2	S	≤ 1	S	64	R	≤ 16	S
*K. oxytoca* #6	-	-	-	-	16	R	4	S	≤ 2	S	≤ 1	S	64	R	32	S
*K. oxytoca* #7	-	-	-	-	16	R	4	S	≤ 2	S	≤ 1	S	64	R	32	S
*K. oxytoca* #8	-	-	-	-	16	R	4	S	≤ 2	S	≤ 1	S	≤ 16	S	32	S
*K. oxytoca* #9	-	-	-	-	8	R	≤ 2	S	≤ 2	S	≤ 1	S	128	R	≤ 16	S
*K. oxytoca* #10	-	-	-	-	16	R	4	S	≤ 2	S	≤ 1	S	≤ 16	S	≤ 16	S
*K. pneumoniae* #11	-	-	SHV61	-	16	R	4	S	≤ 2	S	≤ 1	S	128	R	32	S
*K. oxytoca* #12	-	-	-	-	16	R	4	S	≤ 2	S	2	S	32	S	32	S
*K. oxytoca* #13	-	-	-	-	16	R	4	S	≤ 2	S	≤ 1	S	32	S	≤ 16	S
*K. oxytoca* #14	-	-	-	-	≥ 32	R	8	S	≤ 2	S	≤ 1	S	32	S	≤ 16	S
*E. coli* #15	-	-	-	-	≤ 2	S	≤ 2	S	4	S	4	S	≤ 16	S	≤ 16	S

-: none detected; ^a)^ Detected by PCRs according to Batchelor et al. (2005); Pitout et al. (1998); For all isolates, identical MICs (µg/mL) were obtained with the following substances: Cefotaxime (≤ 1), Ceftazidime (≤ 1), Ceftriaxone (≤ 1), Cefepime (≤ 1), Ertapenem (≤ 0.5), Meropenem (≤ 0,25), Amikacin (≤ 2), Gentamicin (≤ 1), Ciprofloxacin (≤ 0,25), Norfloxacin (≤ 0.5), and Trimethoprim/Sulfamethoxazole (≤ 20); I: Interpretation; S: sensitive; R: resistant; AMP, ampicillin; SAM, ampicillin/sulbactam; CEF, cephalothin; CXM, cefuroxime; FOF, fosfomycin, NIT, Nitrofurantoin; ^g^) The breakpoints to discriminate between susceptible (S), intermediate (I), and resistant (R) were ≤ 1, 2, and ≥ 4 µg/mL for cefotaxime, CTX; ≤4, 8, and ≥ 16 µg/mL for ceftazidime, CAZ; ≤2, 4–8, and ≥ 16 µg/mL for cefepime, CEP; ≤8, 16, and ≥ 32 µg/mL for cefoxitin, COX; ≤1, 2, and ≥ 4 µg/mL for meropenem, MER; ≤0.5, 1, and ≥ 2 µg/mL for ertapenem, ERT (according to CLSI (2020) guidelines, document M100-S23).

### Antimicrobial susceptibility

Besides the β-lactam resistances, the isolates were also tested for resistance to other antibiotics. Antibiotic susceptibility evaluated by MIC showed resistance to ampicillin in 12 (80%) isolates, of which all were *Klebsiell*a spp. In addition, six *Klebsiella* spp. isolates were resistant to fosfomycin. For the remaining antibiotics evaluated, all isolates were sensitive. Resistance to two antimicrobial groups´ ampicillin and fosfomycin was detected only in six *Klebsiella* spp. isolates, including five *K. oxytoca* and one *K. pneumoniae* (Table 2).

### Detection of shiga toxin-producing *E. coli*

All *E. coli* isolates obtained from three bulk-milk samples were negative for both *stx1* and *stx2* genes.

### Evaluation of total bacterial count

The mean value for total bacterial count was 6.97 × 10^6^ CFU/mL (95% CI 6.55 × 10^6^ − 2.05 × 10^7^ CFU/mL) and a geometric mean of 2.16 × 10^4^ CFU/mL (95% CI 1.62 × 10^4^– 28.83 × 10^4^ CFU/mL). A total bacterial count value ≤ 100,000 CFU/mL was found in 84% (126/150) of the herds. According to these results, most of the herds had adequate hygienic quality following Colombian legislation [2].

### Risk factors associated with total bacterial count > 100,000 CFU/mL

The bivariate analysis identified several factors that were significantly associated with the increase in total bacterial count (*p* < 0.25) and were included in the final model: the milker was the owner of the cows (*p* = 0.018), milking site (*p* = 0.022), use of automatic separators (*p* = 0.039), disinfection of the milking system (*p* = 0.212), type of disinfection protocol of the milking machine (*p* = 0.091), adequate washing and disinfection of the milking machine (*p* = 0.142) (Table 3). Concerning the final model, four variables were found to be associated with the risk of presenting CFU > 100,000 CFU/mL: Herds with more than 60 milking cows (RR = 1.35; 95% CI 1.35–1.35), changing milker in the last month (RR = 2.31; IC 95% 1.54–3.47), milking in the paddock (RR = 3.95; IC 95% 1.29–12.09) and use a chlorinated product for pre-dipping (RR = 1.35; IC 95% 1.06–1.73). The variables considered a protective factor to reduce the total bacterial count were the following: when the milker was the owner of the cows (RR = 0.40; IC 95% 0.23–0.71) and *adequate washing and disinfection of the milking machine* (RR = 0.79; IC 95% 0.71–0.87). There was an interaction between changing milker in the last month and *adequate washing and disinfection of the milking machine* (*p* < 0.05) (Table 4).

**Table 3:** Bivariate analysis of the association of factors with the increase of total bacterial count in bulk tank milk samples (*n* = 150) from three municipalities of Antioquia (Colombia)

Variable		Category	total bacterial count > 100.000 CFU/mL			*p*-value
			Observations	%	Total	
**General factors**	Milking cows	< 30	10	14,9	67	0,819
		30–60	10	15,6	64	
		> 60	4	21,1	19	
	The milker is the owner of the cows	No	16	23,9	67	0,018*
		Yes	8	9,6	83	
	changing milkers in the last month	No	19	16,1	118	0,948
		Yes	5	15,6	32	
	Milking system	Mechanical	23	16,7	138	0,531
		Manual	1	11,1	9	
		Both	0	0	3	
	Milking system and location	Mechanical in-parlor	5	7,5	67	0,022*
		Mechanical in-paddock	18	25,4	71	
		Manual in-paddock	1	11,1	9	
		Othersª	0	0	3	
	Automatic separators	With separators	0	0	15	0,039*
		No separators	23	18,7	123	
		Manual milking	1	8,3	12	
**Washing and disinfection of milking equipment and tank**	Milking system disinfection	Automatic	4	8,9	45	0,212*
		Manual	19	19,8	96	
		Not applicable	1	11,1	9	
	Milking machine disinfection protocol	No	0	0	1	0,762
		Yes	23	16,4	140	
		Not applicable	1	11,1	9	
	Protocol type	^b^Formal	10	12	83	0,091*
		^c^Not formal	13	22,8	57	
	Adequate washing and disinfection of the milking machine	No	14	20,9	67	0,142*
		Yes	10	12	83	
	BTM disinfection protocol	No	0	0	1	> 0,99
		Yes	24	16,1	149	
	Protocol type	^b^Formal	12	14,5	83	0,539
		^c^Not formal	12	18,2	66	
	Adequate washing and disinfection of the BTM	No	12	17,9	67	0,566
		Yes	12	14,5	83	
**Milking protocol**	Pre-dipping is performed	No	3	10,7	28	0,57
		Yes	21	17,2	122	
	Pre-dipping solution	Chlorinated	4	15,4	26	0,667
		Iodinated	16	18,8	85	
		Other^d^	1	9,1	11	
	Pre-dipping product concentration	Recommended by the manufacturer	0	0	69	0,451
		Not recommended by the manufacturer	10	14,5	53	
		Not applicable	11	20,8	28	
	Contact time of the teats with the pre-dipping product	< 15 s	3	10,7	75	0,495
		15–30 s	12	16	23	
		> 30 s	6	26,1	24	
		Not applicable	3	12,5	28	

*Significant variables *p* < 0.25; ^a^both systems; ^b^Elaborated by an external provider; ^c^Own elaboration; ^d^ Alcohol, chlorhexidine, and lactic acid

**Table 4:** Final model evaluating the association of some factors with the increase of total count bacteria > 100,000 CFU/mL in bulk tank milk samples (*n* = 150) from three municipalities of Antioquia, Colombia

Variable	RR	SE	Z	*P*>|z|	95% CI
Milking cows					
30–60	1.14	0.503	0.31	0.758	0.48–2.71
> 60	1.35	6.18	6.6	0.000*	1.35–1.35
The milker is the owner of the cows	0.40	0.12	-3,11	0.002*	0.23–0.71
Changing milkers in the last month	2.31	0.48	4.02	0.000*	1.54 - 3.47
Milking In-paddock	3.95	2.25	2.40	0.016*	1.29–12.09
Using automatic separators					
No	2.07	2.21	0.69	0.493	0.26–16.78
Yes	1	(empty)			
Adequate washing and disinfection of the milking machine	0.79	0.039	-4.75	0.000*	0.71–0.87
Pre-dipping solution					
Chlorinated	1.35	0.17	2.43	0.015*	1.06–1.73
Iodinated	1.59	0.51	1.45	0.148	0.85–2.99
Others^a^	1.12	0.31	0.40	0.689	0.65–1.93
Interac4	0.17	0.15	-2.02	0.043*	0.029–0.945
Cons	0.04	0.06	-2.02	0.043*	0.0015–0.905

*Significant variables *p* < 0.05; RR: Risk ratio; SE: standard error; *P*>|z|: *P* value; ^a^Alcohol, chlorhexidine, and lactic acid

## Discussion

A total of 15 bulk tank milk samples, collected from an equal number of dairy farms in three municipalities including Santa Rosa de Osos, San Pedro de los Milagros, and Entrerríos located in Northern Antioquia were confirmed as positive by bacteriological examination. From these samples, one isolate representing each sample/dairy herd, 11 *K. oxytoca*, one *K. pneumonia*, and three *E. coli* were isolated. Notably, among these isolates, only the isolate *K. pneumoniae* #11 was *bla*SHV-61 positive, which was isolated from a dairy farm in the municipality Entrerríos. In the previous study carried out in 2017 exclusively in the single municipality Entrerríos, about 3% of randomly selected dairy herds were found to have ESBL-producing *Enterobacteriaceae* present [17]. The low frequency of BLEE producers determined in this study (< 1%) is not surprising, since it is in agreement with another study carried out in the area [17]; however, there are still few studies on the frequency of BLEE-producing bacteria at the dairy farm level in Colombia that provide absolute clarity on the subject. We hypothesize that in addition to the low use of antibiotics at farm level together with adequate hygiene standards required by local dairy processing plants could be the reason for this favourable situation. Supporting this view, bonuses offered by some milk processing plants for raw milk with total bacterial count values of less than 175,000 CFU/mL stimulate dairy farms in the implementation of adequate hygiene practices at the time of milking, washing equipment, and promote the cold chain, and therefore reduce the occurrence of environmental pathogens and total bacteria values.

On the other hand, the mean total bacterial count was lower than that found previously in Colombia [18] and most herds (84%) had a total bacterial count value ≤ 100,000 CFU/mL, indicating adequate hygienic quality. However, the bacterial count value is still high in 16% of the herds, which indicates the need of improving practices aiming the hygienic quality in the herds. The variables associated with increased bacterial count in raw milk could be explained by the short time the milker has to implement the necessary measures to control quality at milking, the choice of cleaning materials and utensils, the cleaning of the cows, as well as the lack of training of the milkers [19].

The milk samples of the present study were examined using a non-selective method, in which the samples were cultured on non-selective media for the detection of EBSL-producing Enterobacteriaceae and a randomly selected subset of *E. coli* and *Klebsiella* spp. isolates were then subjected to susceptibility testing. It should be noted that ESBL-producing organisms, which might be present as minor constituents of the total bacterial microbiota in tank milk, could potentially go undetected due to the lower sensitivity (10 CFU/mL) of this approach. This stands in contrast to the selective enrichment method which has been previously employed for tank milk samples in various reports [11, 12, 20]; large differences in sensitivity are to be expected between these methods. The results of this study suggest that the sensitivity of this non-selective method for detecting ESBLs might be comparatively lower.

With regard to the ESBL types detected, it was expected to identify clones that harbor the CTX-M gene based on findings of a previous study in the same municipality Entrerríos [17]. Instead, the *bla*SHV gene in a *K. pneumoniae* isolate was identified, suggesting that mobile genetic elements or different types of clones circulate in the study area. Since in the present study, EBSL was detected only in *K. pneumoniae*, it has been reported more frequently in recent years that *K. pneumoniae* can develop multiple mechanisms to become resistant to antibiotics including the production of antibiotic inactivating enzymes such as β-lactamases and ESBLs [21]. This finding is comparable to what has been found in different studies from other countries in bulk-tank milk samples [12, 20, 22], and in milk samples from cows with bovine mastitis [23–25]. In Colombia, ESBL of the SHV type has already been detected in *K. pneumoniae* in clinical patients [26–28], in community environments [29], also in *Salmonella* from retail broiler meat samples [30], and in *E. coli* from different sources in the poultry production [31]. However, the distribution and frequency of ESBL types can vary considerably depending on the source of isolates and geographic variations [32–38].

In the present study, ESBL-producing *K. pneumoniae* was not identified by phenotypic methods, but by molecular methods, which is consistent with previous studies that have reported discrepancies between detection methods [39–41]. The lack of phenotypic resistance in this study could be due to the bacteria was not being subjected to high selection pressure to produce SHV enzyme in the phenotypic confirmation assay. This would indicate that the isolates do not reveal changes in the phenotypic patterns of resistance, unlike what has been evidenced in other reports in which *K. pneumoniae* carrying *bla*SHV presented phenotypic resistance to ampicillin, aztreonam, chloramphenicol, and trimethoprim and inhibitor test sensitivity [37]. This confirms the importance of implementing specific molecular tests for epidemiological monitoring studies due to their high level of specificity [42] since it has been identified that up to 33% of positive strains are not detected with phenotypic methods [43].

Regarding to antibiotic susceptibility of isolates evaluated, *E. coli* isolates obtained were sensitive to all the antibiotics, however, other local studies have shown resistance patterns of *E. coli* isolated from bulk milk samples [17] and from bovine mammary quarter samples [44] in the same region and in other regions of Colombia [45]. In contrast, studies from the same study area on *Klebsiella* spp. isolated from dairy cattle with clinical and subclinical mastitis showed a 40% susceptibility to ampicillin [44], whereas in other countries the rate of resistance to the same antibiotic was variable [23, 46–49]. In addition, the fosfomycin resistance detected in this study is consistent with previous studies reporting *K. pneumoniae* from raw milk [47]. All isolates were susceptible to the ertapenem and meropenem. These findings are not surprising, since commercial preparations containing carbapenems are not approved for use in livestock in Colombia.

Finally, no STEC isolates were found, which relates to the good hygienic quality of raw milk found in most of the herds and the low frequency of *E. coli* isolated considering that not all *E. coli* strains present in bovine feces are STEC [50]. However, epidemiological surveillance programs are still required for food of animal origin since STEC strains can contaminate raw milk through a fecal source [6], or environmental [51].

In conclusion, the frequency of ESBL-producing *E. coli* and *Klebsiella* spp. in bulk-tank milk samples from three municipalities in northern Antioquia was very low (< 1%). Only *K. pneumoniae* was positive for the ESBL type *bla*SHV by sequence analysis. Most dairy herds had adequate hygienic quality and herds with more than 60 milking cows, changing milker, milking in paddocks, and using a chlorinated product for pre-dipping are risk factors that increase the total bacterial count in raw milk. Finally, it was identified that some variables such as the milker being the owner of the animals, and the adequate washing and disinfection of the milking machine promote adequate hygienic milk quality of the raw milk stored in the tanks.

## Data Availability

All data generated or analyzed during this study are included in this published article.
